# Mechanisims of asthma and allergic disease – 1083. CD23, Total IgE and Th1/Th2 cytokines in asthma patients

**DOI:** 10.1186/1939-4551-6-S1-P79

**Published:** 2013-04-23

**Authors:** Anchoju Vijayendra Chary

**Affiliations:** 1 Microbiology and Immunology, National Institute of Nutrition (ICMR), Hyderabad, India

## Background

CD23 (FcεRII), is a low affinity receptor for IgE, likely to influence IgE production and inflammation in allergic diseases. The aim of this study was to determine sCD23 and cytokine levels in asthma patients.

## Methods

Soluble CD23, total histamine release, total IgE and Th1,Th2 cytokines were determined in blood samples of patients with asthma(50) and age and sex matched healthy volunteers (without signs of asthma) (n =20).

## Results

Serum sCD23 mean±SE was significantly increased (p<0.05) in asthma (581.16 ± 35.72 pg/mL), when compared to controls (429.49 ± 31.29 pg/mL). Similarly serum IgE mean±SE (154.03 ± 33.24) and blood histamine (46.7 ± 7.23) levels were increased significantly (P<0.01) in patients with asthma; while IFN-γ, a Th1 cytokine, was significantly lower (P<0.05) in asthma (3.28 ± 0.65) than in controls (9.45 ± 1.58). Pearson's correlation coefficient showed a significant (P<0.05, r=0.50) association between sCD23, IL-5 with serum IgE concentration, however, IFN- γ was not correlated with IgE. Serum IL-4 and CD23 in buccal mucosa and stool samples were below detectable levels.

## Conclusions

Our observations provide evidence on increased CD23 expression in asthma and a preferential activation of Th2 (IL-5) and suppression of Th1 (IFN- γ) response in adults with asthma. Positive correlation between IgE levels and sCD23 was detected in the asthma group.

